# Supplementary material for: Circulating Tumor DNA Profiling of a Diffuse Large B Cell Lymphoma Patient with Secondary Acute Myeloid Leukemia
Source: Cancers (Basel). 2022 Mar 8;14(6):1371. doi: 10.3390/cancers14061371 (PMC8946858; doi:10.3390/cancers14061371)
Supplement: Supplementary file 1 [file cancers-14-01371-s001.zip › Supplemental Methods and Tables.pdf]

Circulating tumor DNA profiling of a diffuse large B cell lymphoma patient with secondary acute myeloid leukemia

Supplemental Methods

Patient and samples

The patient presented here was part of the prospective non-interventional LYMPHCIN study carried out at the University Hospital of the Technical University of Munich (TUM), Germany from 2017–2020. Patient inclusion criteria were: recent diagnosis of non-Hodgkin B cell lymphoma, at least 18 years of age, consent to the study, and no presence of other malignancies at the time of enrolment. The study was conducted in accordance with the Declaration of Helsinki and approved by the Institutional Review Board (Ethics Committee) of the Technical University of Munich (number 47/17 S, March 2017). Written informed consent was obtained from all patients.

Peripheral venous blood samples were taken preferably at first diagnosis or diagnosis of relapse, and then at each follow-up visit to the clinic, which includes the beginning of each treatment cycle, each planned or unplanned visit (such as neutropenic fever) as well as on clinical imaging dates. Blood was collected in one 9 ml EDTA tube. Blood plasma was obtained by centrifugation of blood tubes at 2000 x g for 15 min at 15°C, careful removal of the supernatant without disturbing the buffy coat, additional centrifugation of the supernatant at 2000 x g for 15 min at 15°C, and storage of the supernatant plasma at −80°C.

Histopathological and immunohistochemical diagnosis of an initial DLBCL

The diagnosis of non-GCB type DLBCL of our patient was established in November 2016 by fine-needle aspirate biopsy of para-aortic lymph node masses followed by histopathological morphological as well as immunohistochemical workup. Initial immunohistochemistry staining against CD20, PAX5, CD3, CKpan, S-100, MIB-1 revealed blast cells with a strong positive staining reaction for CD20 and nuclear co-expression of PAX-5. Proliferation rate, visualized with MIB-1, was about 80%. Staining reactions against S-100 and CKpan in the blast cell population were both negative. Additional immunohistochemistry staining against EBV/EBER, cyclin D1, CD30, BCL6, BCL2, CD10, MUM1, and TdT revealed lymphoid

infiltrates with strong positive staining reaction for BCL2, partial nuclear, moderately strong positive staining reaction for BCL6 (about 40%) and strong nuclear co-expression of MUM1. Staining reactions against CD10, cyclin D1, CD30, and TdT were all negative. Negative EBV in situ hybridization. In conclusion, this established the diagnosis of an aggressive non-Hodgkin's lymphoma of the B-cell lineage, in particular of the diffuse large B-cell lymphoma subtype, immunohistochemical non-GC type according to Hans et al. (PMID 14504078).

Hematopathology immunophenotypic diagnosis of a secondary AML

An AML was diagnosed in March 2018 by microscopic and immunophenotyping characterization of peripheral blood. Herein, after red blood cell lysis, in the CD45+ gate 2% monocytes and 96% myeloid cells were found. About 76% myeloid blasts with co-expression of CD117+, CD7+, CD64+, CD4+, CD13+, CD15+, HLA-DR+, CD33+, CD11b+ and absent expression of CD34–, CD56–, CD14–, CD41–, GlyA– were found. In addition, 5% lymphocytes were present. Of these, 81% T cells, 0% B cells and 15% NK cells. In conclusion, this established the diagnosis of an AML by immunophenotypical detection of 76% myeloid CD34– blasts in the peripheral blood.

Targeted sequencing of cell-free plasma DNA

Cell-free-DNA was isolated from stored plasma using the QIAamp Circulating Nucleic Acid Kit (Qiagen, Hilden, Germany) according to the manufacturer’s protocol and quantified on a Qubit 2.0 fluorometer (Thermo Fisher Scientific, San Jose, California). We used the Avenio Expanded Kit (Roche Diagnostics, Mannheim, Germany) comprising 77 genes to prepare a library for targeted next-generation sequencing from 50 ng of plasma DNA according to the manufacturer’s protocol. Libraries were sequenced on a NextSeq 500 system (Illumina, San Diego, USA). Sequencing data were transferred to and analyzed by Roche Diagnostics, Mannheim, Germany. In order to further filter the sequencing data for tumor specific somatic mutations, we used the annotations in the Exome Aggregation Consortium (ExAC) dataset [15] and the COSMIC database [16]. The ExAC frequency represents the individual frequency of a gene variation found in a reference group of ~60,000 healthy individuals. Since allele frequencies above 0.5% are associated with germline inheritance, we focused on all mutations with an ExAC frequency below 0.5%, which are of somatic and thus of potentially relevant nature. Patient specific mutations detected by targeted sequencing of the plasma samples were validated by performing on lymphoma tissue extracted at initial diagnosis. After

detecting patient specific somatic mutations by targeted sequencing in the plasma, tumor specific ddPCR assays for ctDNA quantification were designed.

Variant calling and variant annotation

We used the post-sequencing analysis software “AVENIO Oncology Analysis Software” (Roche Diagnostics, Mannheim, Germany), which is included in the Avenio Expanded Kit to call and annotate mutations from the FASTQ files obtained by sequencing. According to the manufacturer, this software uses the following annotation databases:

Reference Genome: hg38

COSMIC: v83. Catalog of Somatic Mutation in Cancer. (URL: cancer.sanger.ac.uk.) (Sept, 2014). ; Forber S.A. et al. COSMIC: exploring the world's knowledge of somatic mutations in human cancer. Nucl. Acids Res. 43 (D1): D805-D811 (2015).

TCGA: 9.0. The results shown here are in part based upon data generated by the TCGA Research Network: http://cancergenome.nih.gov.

ExAC: 1.0. Exome Aggregation Consortium et al. Analysis of protein-coding genetic variation in 60,706 humans. bioRxiv http://dx.doi.org/10.1101/030338. Roche Sequencing Solutions would like to thank the Exome Aggregation Consortium and the groups that provided exome variant data for comparison. A full list of contributing groups can be found at http://exac.broadinstitute.org/about.

dbSNP: 150. Database of Single Nucleotide Polymorphisms (dbSNP). Bethesda (MD): National Center for Biotechnology Information, National Library of Medicine. (dbSNP Build ID: 150). Available from: http://www.ncbi.nlm.nih.gov/SNP/.

1000 Genomes: phase_3_v5b. The 1000 Genomes Project Consortium. A global reference for human genetic variation. Nature. 526 (7571), 68-74 (2015).

SnpEff: 4.2. Cingolani P. et al. A program for annotating and predicting the effects of single nucleotide polymorphisms, SnpEff: SNPs in the genome of Drosophila melanogaster strain w1118; iso-2; iso-3. Fly (Austin). 6(2), 80-92 (2012).

Mutational profiling of cell-free plasma DNA using droplet digital PCR

Droplet digital PCR (ddPCR) primer pairs and probes were designed for six somatic mutations as well as their wild-type equivalent. Assays were validated using positive controls (gBlocks, synthetic DNA with the amplicon sequence containing the mutation or containing wildtype, obtained from Integrated DNA Technologies, Coralville, Iowa, United States), NTC (purified, nuclease-free water), and negative controls (10 ng gDNA, genomic DNA from peripheral blood leukocytes of healthy subjects). These controls were also included for each assay run. Samples were run on a QX200 Droplet Digital PCR (ddPCR) system with automated droplet generation (Bio-Rad Laboratories, Hercules, California, United States). Reactions were carried out in ddPCR 96-well plates (#12001925, Bio-Rad). Each well contained 10.5 µl of ddPCR supermix for probes (no dUTP; Bio-Rad), 1.05 µl of target-specific primers (900 nmol/l), 1.05 µl of target-specific probe (250 nmol/l), 1.05 µl of water, and 6.3 µl of sample DNA, for a total volume of 21 µl. Plates were sealed, spun down and loaded into the droplet generator. Immediately after droplet generation, 96-well plates containing droplet-partitioned samples were sealed and PCR was carried out on a C1000 Touch Thermal Cycler (Bio-Rad) using the following cycling protocol: enzyme activation at 95°C for 10 minutes, followed by 40 cycles of 94°C for 30 seconds (for denaturation) and 55°C for 60 seconds (for annealing/extension), followed by a final 10-minute incubation at 98°C (for enzyme deactivation), with a ramp rate of 2°C per second. Plates were then kept at 4°C. All samples were measured in duplicates (two wells). Finally, plates were read on a QX200 droplet reader (Bio-Rad) to determine droplet fluorescence intensities.

Droplet digital PCR data analysis

Raw droplet fluorescence intensity values were exported from QuantaSoft droplet reader software v1.7.4 (Bio-Rad). Custom scripts were used to import the intensity values into R (version 3.4.4; http://www.r-project.org) and to quantify concentrations of mutant and wildtype DNA. Target concentrations c were calculated for each well from the number of positive droplets Np and negative droplets Nn and the average droplet volume V = 0.85 nanoliter based on Poisson distribution statistics using the formula $c = (\ln(Np + Nn) - \ln(Nn))/V$, where ln is the natural logarithm.

Supplemental Tables

Supplemental Table S1 -- ctDNA

Supplemental Table S1. Mutations identified by targeted sequencing of plasma DNA.

Results of the targeted next-generation sequencing of four plasma cfDNA samples with the Avenio Expanded Kit.

Supplemental Table S2 -- ctDNA

Supplemental Table S2. Genes and regions targeted in the Avenio Expanded Kit.
